# Transcutaneous Vagus Nerve Stimulation: A Promising Method for Treatment of Autism Spectrum Disorders

**DOI:** 10.3389/fnins.2016.00609

**Published:** 2017-01-20

**Authors:** Yu Jin, Jian Kong

**Affiliations:** ^1^Department of Maternal and Child Health, School of Public Health, Sun Yat-sen UniversityGuangzhou, China; ^2^Department of Psychiatry, Massachusetts General Hospital, Harvard Medical SchoolCharlestown, MA, USA

**Keywords:** Autism Spectrum Disorder, transcutaneous electric nerve stimulation, ear, vagus nerve, transcutaneous vagus nerve stimulation, Non-invasive vagus nerve stimulation

## Abstract

Transcutaneous Vagus Nerve Stimulation (tVNS) on the auricular branch of the vagus nerve has been receiving attention due to its therapeutic potential for neuropsychiatric disorders. Although the mechanism of tVNS is not yet completely understood, studies have demonstrated the potential role of vagal afferent nerve stimulation in the regulation of mood and visceral state associated with social communication. In addition, a growing body of evidence shows that tVNS can activate the brain regions associated with Autism Spectrum Disorder (ASD), trigger neuroimmune modulation and produce treatment effects for comorbid disorders of ASD such as epilepsy and depression. We thus hypothesize that tVNS may be a promising treatment for ASD, not only for comorbid epilepsy and depression, but also for the core symptoms of ASD. The goal of this manuscript is to summarize the findings and rationales for applying tVNS to treat ASD and propose potential parameters for tVNS treatment of ASD.

## Introduction

Autism Spectrum Disorders (ASD) refers to a group of lifelong neurodevelopmental disorders, characterized by persistent deficits in social communication and restricted, repetitive behavior (McPartland et al., 2012). The prevalence of ASD has rapidly increased to over 2% and has become a significant public health concern (Kim et al., 2013; Zablotsky et al., 2014). Currently, the etiology of ASD remains unclear, and there is still no targeted treatment. Thus, there is an urgent need to develop new therapies for ASD.

Here, we propose transcutaneous vagus nerve stimulation (tVNS) on the ear as a promising method for treatment of ASD. The vagus nerve consists of a complex network that regulates one's neuro-endocrine-immune axis mood, pain, and memory (Yuan and Silberstein, 2016). It serves as a control center that integrates interoceptive information and responds with appropriate adaptive modulatory feedbacks (Yuan and Silberstein, 2016).

Vagus Nerve Stimulation (VNS) has been used for more than 20 years as an non-pharmacological treatment epilepsy depression (George et al., 2000; Kosel and Schlaepfer, 2002; Daban et al., 2008; Yuan and Silberstein, 2016). VNS requires surgical implantation of a bipolar electrode around the left cervical vagus nerve and a pulse generator under the skin of the left chest (George et al., 2000). Intermittent electrical signals sent from the generator at a low frequency stimulate the cervical vagus nerve and conduct to various regions of the brain (George et al., 2000). After the efficacy and safety of VNS was verified, VNS was approved by the US Food and Drug Administration for treatment-resistant epilepsy in 1997 and for treatment-resistant depression in 2005. The main side-effects of VNS in patients include surgical complications, dyspnea, parasthesia, headache, pain, pharyngitis, hoarseness, cough, and throat discomfort (Ramsay et al., 1994; Ben-Menachem et al., 2015). The precise mechanism of VNS for treatment is still unknown. It was believed that the vagus nerve is linked to brain regions involved in mood regulation, such as the prefrontal cortex and amygdala which can produce antidepression effect (George et al., 2000; Drevets et al., 2002).

The surgical risks and potentially significant side effects have limited this treatment's use in patients with Major Depressive Disorder who have been treated for depression but failed to respond to at least 4 prescribed medications and/or tried somatic treatment options such as electroconvulsive therapy (Daban et al., 2008). The therapeutic potential of VNS has led to the development of non-invasive VNS, which greatly reduces the risks associated with the old procedure (Ben-Menachem et al., 2015). The tVNS is a safe, non-invasive, and low-cost method modified from VNS (Kraus et al., 2007; Dietrich et al., 2008; Kreuzer et al., 2012). The rationale of tVNS at the ear is that anatomical studies suggest that the ear is the only location on the surface of the human body where there is afferent vagus nerve distribution (Peuker and Filler, 2002). According to the “bottom-up” mechanism of the central nervous system, the propagation of electric stimuli might follow an inverse path from peripheral nerves toward the brain stem and central structures to produce therapeutic effect (Shiozawa et al., 2014).

The support for applying tVNS as a treatment for ASD can be summarized in four points: (1) Impaired social-emotional communication skills and repetitive behavior are two core symptoms in ASD from very early childhood to adulthood. The vagus nerve is a key component in regulating the autonomic nervous system, social-emotional function, and adaptive behavior. Investigators found that VNS may have positive social-emotional effects independent of seizure control in patients with intractable epilepsy and ASD (Porges, 1997; Murphy et al., 2000; Levy et al., 2010; Hull et al., 2015). (2) Studies have suggested that abnormally functioning connections within and between the frontal, temporal and parietal cortices, and subcortical structure (thalamus, amygdala, and hippocampus) are involved in the social dysfunction and repetitive behavior seen in ASD (Cheng et al., 2015). Vagal stimulation can modulate the cortical and subcortical (particularly the amygdala and thalamus) functions, which may in turn regulate the disturbed brain function of ASD (Kraus et al., 2007; Frangos et al., 2015). (3) Disturbed immune function is frequently observed in ASD individuals, the hypothesis of altered immune response in ASD has been proposed (Gesundheit et al., 2013). Studies suggest that stimulation of the vagus nerve could downregulates inflammatory cytokine release (Lerman et al., 2016) (4) tVNS could treat the comorbidities of ASD, such as epilepsy(Park, 2003) and depression (Fang et al., 2016; Rong et al., 2016). In addition, studies suggest that the pathogenesis of ASD, epilepsy and depression may overlap, (Tuchman and Rapin, 2002) and tVNS might have a treatment effect on a common pathway of these three disorders (Lulic et al., 2009; Fang et al., 2016).

**Figure 1:**
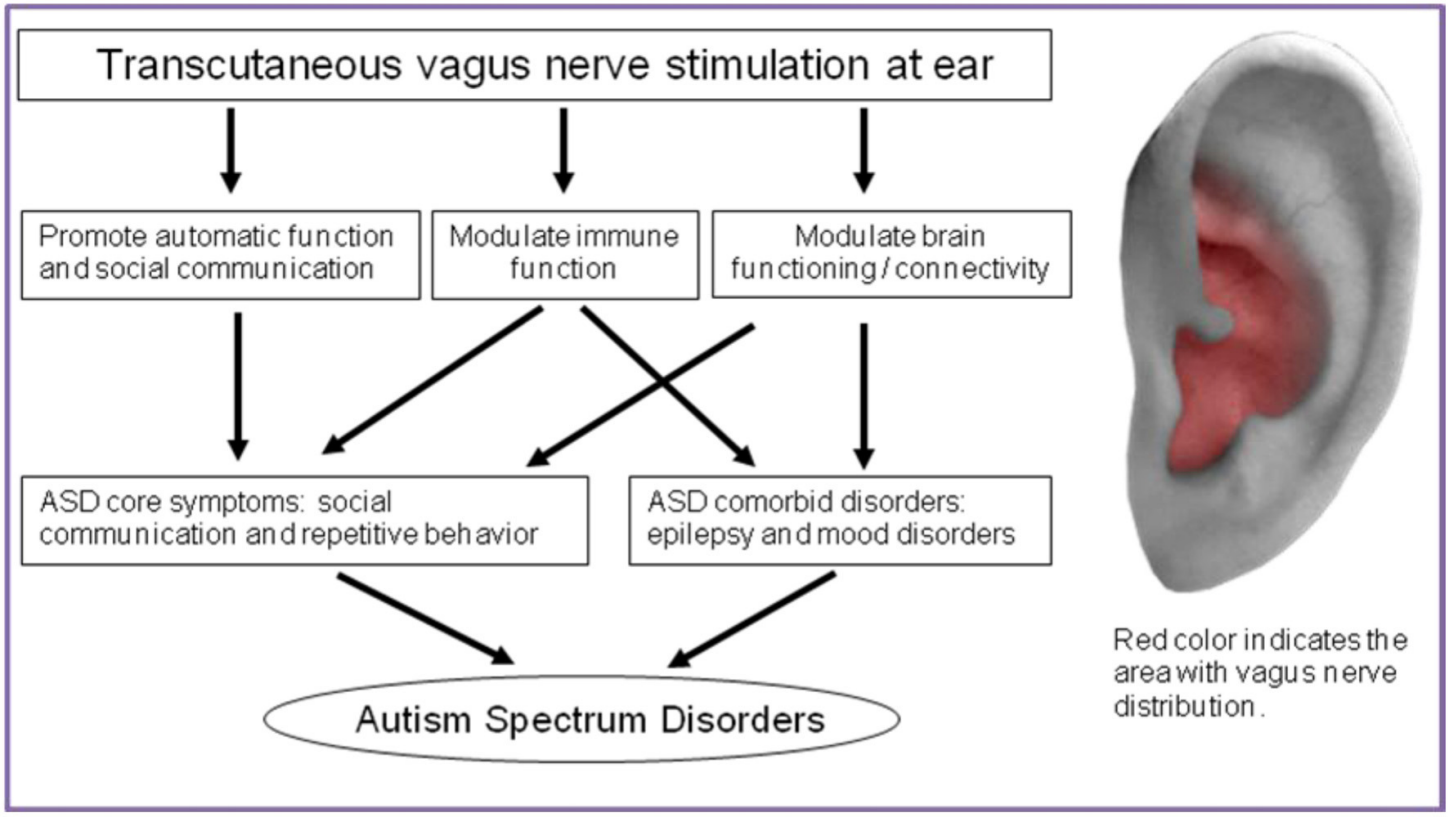
**Action mechanism of tVNS on treatment for ASD and the location of vagus nerve distribution in ear**.

Based on the above evidence, we propose that tVNS should be included as a treatment option for ASD (Figure 1). The aim of this manuscript is to summarize the findings and mechanisms of tVNS treatment for ASD, and propose potential parameters for its application.

## tVNS can modulate the core functions impaired in ASD

Vagus nerve activity can modulate emotional social interactions and repetitive behavior of children (Porges, 1997; Levy et al., 2010; Hull et al., 2015). According to the social engagement system model, a high level of vagal activity is associated with better social skills, such as synchronous mother-infant interactions and positive affect (Porges, 2003). Low vagal activity is associated with less vocalizing (Porges, 2001). During social engagement, facial expressions (cranial nerve VII), listening (cranial nerve VIII), and vocalization (cranial nerves IX, X) can be integrated by vagus nerve activity via branches of the vagus nerve that regulate facial, palpebral, middle-ear, laryngeal, pharyngeal, and mastication muscles and several visceral organ muscles (Porges, 2001).

Clinical observation and studies have shown that children with ASD often present characteristics of low vagal activity, such as flat facial expressions and intonation, (Yirmiya et al., 1989; Chan and To, 2016) difficulties with vocalizing and speech perception, decreased response to their own name and social auditory information, and low baseline cardiac parasympathetic activity (Porges, 2001; Ming et al., 2005; Cygan et al., 2014). Some of these characteristics, such as low vagal tone and expressivity, could be improved by therapies such as repetitive transcranial magnetic stimulation and massage, which can increase vagal activity (Cullen et al., 2005; Diego et al., 2007; Casanova et al., 2014). For instance, touch therapy in form of massage has been shown to improve social response and the relationship between parents and children with Autism (Cullen et al., 2005).

Although still under debate (Danielsson et al., 2008), several studies have shown that there are positive effects on behavior, emotion, and social skills independent of seizure control after VNS on individuals with ASD and epilepsy (Park, 2003; Warwick et al., 2007; Levy et al., 2010; Hull et al., 2015).

For instance, Hull et al. reported that a steady decline of stereotype frequency and aggressive behaviors was evident after one year of VNS treatment in the case of a 10-year-old boy with ASD and comorbid intractable epilepsy and pediatric autoimmune neuropsychiatric disorder associated with streptococcal infections (Hull et al., 2015). In addition, the authors also found there was improvement in following instructions, which is an index of receptive communication skills. All these positive effects are independent of seizure control (Hull et al., 2015). In another case study, Warwick et al. reported a case of a 23-year-old male patient diagnosed with both Asperger syndrome and bitemporal epilepsy who received VNS therapy (Warwick et al., 2007). Both the seizure severity and behavioral components of his Asperger syndrome were improved after monitoring for 6 months (Warwick et al., 2007).

Levy et al. used registered data of VNS therapy outcomes of 315 patients with intractable epilepsy but without ASD and 77 patients with both intractable epilepsy and a diagnosis of ASD (Levy et al., 2010). They found that patients with epilepsy and ASD achieved more quality-of-life improvements in mood subscale at 12 months of post-implant VNS. Park et al. also used a registered system of VNS patients' outcomes and collected data for 6 Landau–Kleffner syndrome patients and 59 autistic patients with intractable seizures (Park, 2003). Landau–Kleffner syndrome is also known as an acquired epileptic aphasia or aphasia with convulsive disorder. The results showed that VNS therapy improved the quality of life of patients with either Landau–Kleffner syndrome or autism comorbid epilepsy, and the improvement in quality of life was independent of its effects on seizures (Park, 2003).

In addition, VNS and tVNS can improve verbal and cognition functions, the development of which is delayed in ASD (Parker et al., 1999; Steenbergen et al., 2015). Parker et al. found that VNS can increase the verbal performance of patients with epileptic encephalopathies independently from seizure frequency improvement (Parker et al., 1999). Additional benefits of VNS therapy include improvement in executive functions, e.g., logical reasoning, response inhibition or impulsiveness, and memory (Clark et al., 1999; Sackeim et al., 2001). Taken together, above results suggest that increasing vagal activity may improve the social and cognitive functions of ASD patients, and provide support for applying VNS therapy on ASD. However, studies of the effectiveness of the tVNS are still mission.

## tVNS can modulate the brain network associated with the neuropathology of ASD

Studies suggested that, the nucleus of the solitary tract is the key target of afferent vagal inputs, and projects toward the locus coeruleus, raphe nuclei, thalamus, amygdala, hippocampus, and neocortex (Kraus et al., 2007; Frangos et al., 2015). Animal studies have verified that vagal stimulation might induce changes in extracellular concentrations of noradrenaline and serotonin in the amygdala, hippocampus, and cortex (Roosevelt et al., 2006; Ruffoli et al., 2011). Ben-Menachem et al. found increased gamma aminobutyric acid (GABA) levels and decreased glutamate levels in the cerebrospinal fluid after 9-months of tVNS treatment on patients with epilepsy (Ben-Menachem et al., 1995). Capone et al. used paired-pulse transcranial magnetic stimulation during tVNS on healthy volunteers and recorded increased GABAergic motor cortical activity, which could be influenced by noradrenergic and serotonergic innervation through the transmitter ascending system from the brainstem (Korchounov et al., 2003; Capone et al., 2015). These studies suggest that vagal stimulation can induce the neuronal activities of noradrenaline, serotonin, and GABA, which may in turn lead to changes in cortical and subcortical activity.

Functional neuroimaging and neurochemical studies have also shown that tVNS on the afferent branch of vagus nerve can activate multiple brain areas involved in social and emotional regulation (Manta et al., 2009; Polak et al., 2009; Frangos et al., 2015). These activation patterns evoked by tVNS are similar to brain activity changes evoked by traditional VNS (Chae et al., 2003; Kraus et al., 2013; Frangos et al., 2015; Fang et al., 2016).

Additionally, a convergence of evidence points to abnormal brain connectivity in ASD (Wass, 2011; Washington et al., 2014). Resting state functional magnetic resonance imaging (fMRI) in ASD showed decreased cortical intrinsic functional connectivity, especially in primary parietal sensorimotor regions, and hyperconnectivity between subcortical regions (especially the thalamus and globus pallidus) (Di Martino et al., 2014). Using resting state fMRI data from a relatively large sample of 418 autism patients, Cheng et al. found reduced cortical functional connectivity between regions associated with facial expression processing, and emotional and social communication, including the ventromedial prefrontal cortex, inferior temporal gyrus, middle temporal gyrus, and superior temporal sulcus. In addition, they also found reduced functional connectivity among brain regions involved in spatial functions and recognition of self and spatial environment (precuneus, superior parietal lobule region) (Cheng et al., 2015).

In parallel, research has provided evidence for the modulation of brain function by tVNS. In a previous study, we found that after 1 month of tVNS treatment in depression patients, the resting state functional connectivity (rsFC) between the default mode network and anterior insula and parahippocampus decreased; the rsFC between the default mode network and precuneus and orbital prefrontal cortex increased compared with sham tVNS (Fang et al., 2016). In another study, we found that the rsFC in the tVNS group between the right amygdala and left dorsolateral prefrontal cortex significantly increased compared with sham tVNS in patients with depression. All the rsFC increases were also associated with Hamilton Depression Rating Scales scores' reduction as well as reductions in the anxiety and retardation subscales (Liu et al., 2016). These studies provide direct evidence on the modulation effect of tVNS on rsFC in patient population. Nevertheless, there was no direct evidence of tVNS effects on central nervous system changes in ASD, further research is needed.

## tVNS can modulate the immune function which is believed to be included in the etiologic hypothesis of ASD

Accumulating evidence suggests that ASD is associated with impaired immune function at both systemic and cellular levels (Heuer et al., 2008; Masi et al., 2015). These neuroimmune abnormalities start from a very early stage of development and continue through the whole lifespan, which may influence neurodevelopment and nervous system function (Heuer et al., 2008; Krakowiak et al., 2015). Studies have verified that dysregulation of the immune system in ASD, and changes in immune function may impact some neurological processes in embryogenesis, including ongoing inflammation in the brain, elevated proinflammatory cytokine profiles in the cerebrospinal fluid and blood, increased presence of brain-specific auto-antibodies and altered immune cell function (Li et al., 2009; Ashwood et al., 2011). ASD patients have high levels of glutamate in the central nervous system, which might interact with immune function (El-Ansary and Al-Ayadhi, 2014). Furthermore, these dysfunctional immune responses are associated with increased impairments in behaviors characteristic of the core features of ASD, in particular, deficits in social interaction and communication (Ashwood et al., 2011; Onore et al., 2012). These evidences suggest that immune processes and neuro-immune interaction play a key role in the pathophysiology of ASD. Accordingly, the hypothesis of immune dysfunction in ASD has been proposed for decades.

Both VNS and tVNS can modulate immune function by a defense response and the top-down modulation mechanism (Zhao et al., 2011; Bonaz et al., 2013). An animal study showed that electrical stimulation of the efferent vagus nerve inhibits the systemic inflammatory response to endotoxin (lipopolysaccharide) administration through the release of the vagal neurotransmitter acetylcholine (Borovikova et al., 2000). Lerman et al. found that VNS might downregulates inflammatory cytokine release, providing evidence for its anti-inflammatory effect (Lerman et al., 2016). Thus, tVNS might modulate not only the immune function of ASD, but also the interaction between neural plasticity and the immune system (Garay and McAllister, 2010).

## Parameters of tVNS for treatment of ASD

Given that the right vagal nerve projects efferent fibers to the heart, investigators have suggested that VNS on the neck to the cervical vagus nerve is safer on the left side of the body. Since there are no direct fibers connecting the ear vagus nerve to the heart (Sperling et al., 2010; Kreuzer et al., 2012) both left and right ears should be safe for applying tVNS.

There are wholly afferent vagus nerve innervations in the Cymba Conchae of the ear, and they mix with the great auricular nerve and auriculotemporal nerve in the Crus of helix, Antihelix, Tragus and Cavity of concha (Figure 1) (Peuker and Filler, 2002). Theoretically, the optimal position for tVNS is the cymba conchae, followed by the other positions mentioned above.

In a recent study, investigators compared fMRI signal changes evoked by tVNS at the anterior and posterior sides of the left outer auditory canal. The results showed that fMRI signal changes were in the opposite direction (activation vs. deactivation) in many brain regions with the exception of the insular cortex (positive blood oxygenation level dependent changes in both conditions) (Kraus et al., 2013). Prominent decreases of the blood oxygenation level dependent signals were detected in the parahippocampal gyrus, posterior cingulate cortex and right thalamus (pulvinar) following anterior auditory canal wall stimulation (Kraus et al., 2013). These results suggest that tVNS at different locations of the ear may modulate different brain pathways, more studies are needed to identify the optimal tVNS location for ASD.

Very few studies have systemically investigated the optimal frequency of tVNS. Previous studies have suggested that different parameters of stimulation during VNS or tVNS could produce different brain changes and neurotransmitter releases. A recent tVNS study in migraine patients showed that although both 1 and 25 Hz tVNS can improve clinical outcome in patients with chronic migraine, 1 Hz tVNS can produce greater improvement than 25 Hz tVNS (Straube et al., 2015). Studies have suggested that a frequency between 1 and 30 Hz, pulse width of 130–1000 μs and intensity of 4–6 mA is sufficient to elicit a therapeutic effect (Fang et al., 2016; Rong et al., 2016). Therefore, the suggested parameters for tVNS treatment of ASD may set a frequency between 1 and 30 Hz, or altered frequency between 1 and 30 Hz.

Likewise, there is no systemic study on the intensity of tVNS. Previous studies suggested that stimulation intensity could be set to a level that could arouse a tingling but tolerable sensation (Rong et al., 2016). More research is needed to explore the optimal tVNS parameters for different subgroups of ASD.

tVNS is a fairly safe treatment method. The reported mild/moderate side effects include local problems at stimulation sites, such as pain, paresthesia, or pruritus during or after stimulation; erythema, ulcers or scabs, (Straube et al., 2015) and tinnitus (Rong et al., 2016). One potential concern for tVNS is its long-term cardiac safety. But in a recent study published in Frontiers in Psychiatry, Kreuzer et al. measured EKG changes after 24 months of tVNS and found that tVNS has no arrhythmic effects on cardiac function in tinnitus patients with no known pre-existing cardiac pathology (Kreuzer et al., 2012). This study endorsed the safety of long-term application of the tVNS.

In summary, tVNS may be a promising method for treating ASD. It holds the potential to not only relieve core symptoms of ASD, but also comorbidities of ASD such as epilepsy, depression, and anxiety. As a non-invasive, low-cost and convenient method with no side-effects on heart rate and blood pressure or peripheral microcirculation, tVNS is a promising treatment option for ASD (Kraus et al., 2007; Frangos et al., 2015).

## Author contributions

YJ: Literature review and manuscript preparation. JK: Conceived the idea and revised this manuscript.

## Funding

YJ was supported by National Natural Science Foundation of China (81171293) and China Scholarship (201506385026), JK was supported by R01AT006364, R01AT008563, R21AT008707, R61AT009310, and P01 AT006663 from NIH/CCIH.

### Conflict of interest statement

The authors declare that the research was conducted in the absence of any commercial or financial relationships that could be construed as a potential conflict of interest.

## Abbreviations

tVNS: transcutaneous vagus nerve stimulation
ASD: autism spectrum disorders
VNS: vagus nerve stimulation
GABA: gamma aminobutyric acid
fMRI: functional magnetic resonance imaging
rsFC: resting state functional connectivity.

## References

[B1] Ashwood P. Krakowiak P. Hertz-Picciotto I. Hansen R. Pessah I. Van de Water J. (2011). Elevated plasma cytokines in autism spectrum disorders provide evidence of immune dysfunction and are associated with impaired behavioral outcome. Brain Behav. Immun. 25, 40–45. 10.1016/j.bbi.2010.08.003 20705131 PMC2991432

[B2] Ben-Menachem E. Hamberger A. Hedner T. Hammond E. J. Uthman B. M. Slater J.. (1995). Effects of vagus nerve stimulation on amino acids and other metabolites in the CSF of patients with partial seizures. Epilepsy Res. 20, 221–227. 10.1016/0920-1211(94)00083-9 7796794

[B3] Ben-Menachem E. Revesz D. Simon B. J. Silberstein S. (2015). Surgically implanted and non-invasive vagus nerve stimulation: a review of efficacy, safety and tolerability. Eur. J. Neurol. 22, 1260–1268. 10.1111/ene.12629 25614179 PMC5024045

[B4] Bonaz B. Picq C. Sinniger V. Mayol J. F. Clarençon D. (2013). Vagus nerve stimulation: from epilepsy to the cholinergic anti-inflammatory pathway. Neurogastroenterol. Motil. 25, 208–221. 10.1111/nmo.12076 23360102

[B5] Borovikova L. V. Ivanova S. Zhang M. Yang H. Botchkina G. I. Watkins L. R.. (2000). Vagus nerve stimulation attenuates the systemic inflammatory response to endotoxin. Nature 405, 458–462. 10.1038/35013070 10839541

[B6] Capone F. Assenza G. Di Pino G. Musumeci G. Ranieri F. Florio L.. (2015). The effect of transcutaneous vagus nerve stimulation on cortical excitability. J. Neural Transm. 122, 679–685. 10.1007/s00702-014-1299-7 25182412

[B7] Casanova M. F. Hensley M. K. Sokhadze E. M. El-Baz A. S. Wang Y. Li X.. (2014). Effects of weekly low-frequency rTMS on autonomic measures in children with autism spectrum disorder. Front. Hum. Neurosci. 8:851. 10.3389/fnhum.2014.00851 25374530 PMC4204613

[B8] Chae J. H. Nahas Z. Lomarev M. Denslow S. Lorberbaum J. P. Bohning D. E.. (2003). A review of functional neuroimaging studies of vagus nerve stimulation (VNS). J. Psychiatr. Res. 37, 443–455. 10.1016/S0022-3956(03)00074-8 14563375

[B9] Chan K. K. To C. K. (2016). Do individuals with high-functioning autism who speak a tone language show intonation deficits? J. Autism Dev. Disord. 46, 1784–1792. 10.1007/s10803-016-2709-5 26825662

[B10] Cheng W. Rolls E. T. Gu H. Zhang J. Feng J. (2015). Autism: reduced connectivity between cortical areas involved in face expression, theory of mind, and the sense of self. Brain 138(Pt 5), 1382–1393. 10.1093/brain/awv051 25795704 PMC4407191

[B11] Clark K. B. Naritoku D. K. Smith D. C. Browning R. A. Jensen R. A. (1999). Enhanced recognition memory following vagus nerve stimulation in human subjects. Nat. Neurosci. 2, 94–98. 10.1038/4600 10195186

[B12] Cullen L. A. Barlow J. H. Cushway D. (2005). Positive touch, the implications for parents and their children with autism: an exploratory study. Complement. Ther. Clin. Pract. 11, 182–189. 10.1016/j.ctcp.2004.12.004 16005835

[B13] Cygan H. B. Tacikowski P. Ostaszewski P. Chojnicka I. Nowicka A. (2014). Neural correlates of own name and own face detection in autism spectrum disorder. PLoS ONE 9:e86020. 10.1371/journal.pone.0086020 24465847 PMC3899112

[B14] Daban C. Martinez-Aran A. Cruz N. Vieta E. (2008). Safety and efficacy of Vagus Nerve Stimulation in treatment-resistant depression. A systematic review. J. Affect. Disord. 110, 1–15. 10.1016/j.jad.2008.02.012 18374988

[B15] Danielsson S. Viggedal G. Gillberg C. Olsson I. (2008). Lack of effects of vagus nerve stimulation on drug-resistant epilepsy in eight pediatric patients with autism spectrum disorders: a prospective 2-year follow-up study. Epilepsy Behav. 12, 298–304. 10.1016/j.yebeh.2007.10.007 18053767

[B16] Diego M. A. Field T. Hernandez-Reif M. Deeds O. Ascencio A. Begert G. (2007). Preterm infant massage elicits consistent increases in vagal activity and gastric motility that are associated with greater weight gain. Acta Paediatr. 96, 1588–1591. 10.1111/j.1651-2227.2007.00476.x 17888059

[B17] Dietrich S. Smith J. Scherzinger C. Hofmann-Preiss K. Freitag T. Eisenkolb A.. (2008). [A novel transcutaneous vagus nerve stimulation leads to brainstem and cerebral activations measured by functional MRI]. Biomed. Tech. 53, 104–111. 10.1515/BMT.2008.022 18601618

[B18] Di Martino A. Yan C. G. Li Q. Denio E. Castellanos F. X. Alaerts K.. (2014). The autism brain imaging data exchange: towards a large-scale evaluation of the intrinsic brain architecture in autism. Mol. Psychiatry 19, 659–667. 10.1038/mp.2013.78 23774715 PMC4162310

[B19] Drevets W. C. Price J. L. Bardgett M. E. Reich T. Todd R. D. Raichle M. E. (2002). Glucose metabolism in the amygdala in depression: relationship to diagnostic subtype and plasma cortisol levels. Pharmacol. Biochem. Behav. 71, 431–447. 10.1016/S0091-3057(01)00687-6 11830178

[B20] El-Ansary A. Al-Ayadhi L. (2014). GABAergic/glutamatergic imbalance relative to excessive neuroinflammation in autism spectrum disorders. J. Neuroinflammation 11:189. 10.1186/s12974-014-0189-0 25407263 PMC4243332

[B21] Fang J. Rong P. Hong Y. Fan Y. Liu J. Wang H.. (2016). Transcutaneous vagus nerve stimulation modulates default mode network in major depressive disorder. Biol. Psychiatry 79, 266–273. 10.1016/j.biopsych.2015.03.025 25963932 PMC4838995

[B22] Frangos E. Ellrich J. Komisaruk B. R. (2015). Non-invasive access to the vagus nerve central projections via electrical stimulation of the external ear: fMRI evidence in humans. Brain Stimul. 8, 624–636. 10.1016/j.brs.2014.11.018 25573069 PMC4458242

[B23] Garay P. A. McAllister A. K. (2010). Novel roles for immune molecules in neural development: implications for neurodevelopmental disorders. Front. Synaptic Neurosci. 2:136. 10.3389/fnsyn.2010.00136 21423522 PMC3059681

[B24] George M. S. Sackeim H. A. Rush A. J. Marangell L. B. Nahas Z. Husain M. M.. (2000). Vagus nerve stimulation: a new tool for brain research and therapy. Biol. Psychiatry 47, 287–295. 10.1016/S0006-3223(99)00308-X 10686263

[B25] Gesundheit B. Rosenzweig J. P. Naor D. Lerer B. Zachor D. A. Prochazka V.. (2013). Immunological and autoimmune considerations of Autism Spectrum Disorders. J. Autoimmun. 44, 1–7. 10.1016/j.jaut.2013.05.005 23867105

[B26] Heuer L. Ashwood P. Schauer J. Goines P. Krakowiak P. Hertz-Picciotto I.. (2008). Reduced levels of immunoglobulin in children with autism correlates with behavioral symptoms. Autism Res. 1, 275–283. 10.1002/aur.42 19343198 PMC2663897

[B27] Hull M. M. Madhavan D. Zaroff C. M. (2015). Autistic spectrum disorder, epilepsy, and vagus nerve stimulation. Child's Nerv. Syst. 31, 1377–1385. 10.1007/s00381-015-2720-8 25922052

[B28] Kim Y. S. Leventhal B. L. Koh Y. J. Fombonne E. Laska E. Lim E. C.. (2013). Prevalence of autism spectrum disorders in a total population sample. Am. J. Psychiatry 168, 904–912. 10.1176/appi.ajp.2011.10101532 21558103

[B29] Korchounov A. Ilic T. V. Ziemann U. (2003). The alpha2-adrenergic agonist guanfacine reduces excitability of human motor cortex through disfacilitation and increase of inhibition. Clin. Neurophysiol. 114, 1834–1840. 10.1016/S1388-2457(03)00192-5 14499745

[B30] Kosel M. Schlaepfer T. E. (2002). Mechanisms and state of the art of vagus nerve stimulation. J. ECT 18, 189–192. 10.1097/00124509-200212000-00004 12468993

[B31] Krakowiak P. Goines P. E. Tancredi D. J. Ashwood P. Hansen R. L. Hertz-Picciotto I.. (2015). Neonatal cytokine profiles associated with autism spectrum disorder. Biol. Psychiatry. [Epub ahead of print]. 10.1016/j.biopsych.2015.08.007. 26392128 PMC4753133

[B32] Kraus T. Hösl K. Kiess O. Schanze A. Kornhuber J. Forster C. (2007). BOLD fMRI deactivation of limbic and temporal brain structures and mood enhancing effect by transcutaneous vagus nerve stimulation. J. Neural Transm. 114, 1485–1493. 10.1007/s00702-007-0755-z 17564758

[B33] Kraus T. Kiess O. Hösl K. Terekhin P. Kornhuber J. Forster C. (2013). CNS BOLD fMRI effects of sham-controlled transcutaneous electrical nerve stimulation in the left outer auditory canal - a pilot study. Brain Stimul. 6, 798–804. 10.1016/j.brs.2013.01.011 23453934

[B34] Kreuzer P. M. Landgrebe M. Husser O. Resch M. Schecklmann M. Geisreiter F.. (2012). Transcutaneous vagus nerve stimulation: retrospective assessment of cardiac safety in a pilot study. Front. Psychiatry 3:70. 10.3389/fpsyt.2012.00070 22891061 PMC3413045

[B35] Lerman I. Hauger R. Sorkin L. Proudfoot J. Davis B. Huang A.. (2016). Noninvasive transcutaneous vagus nerve stimulation decreases whole blood culture-derived cytokines and chemokines: a randomized, blinded, healthy control pilot trial. Neuromodulation 19, 283–290. 10.1111/ner.12398 26990318

[B36] Levy M. L. Levy K. M. Hoff D. Amar A. P. Park M. S. Conklin J. M.. (2010). Vagus nerve stimulation therapy in patients with autism spectrum disorder and intractable epilepsy: results from the vagus nerve stimulation therapy patient outcome registry. J. Neurosurg. Pediatr. 5, 595–602. 10.3171/2010.3.PEDS09153 20515333

[B37] Li X. Chauhan A. Sheikh A. M. Patil S. Chauhan V. Li X. M.. (2009). Elevated immune response in the brain of autistic patients. J. Neuroimmunol. 207, 111–116. 10.1016/j.jneuroim.2008.12.002 19157572 PMC2770268

[B38] Liu J. Fang J. Wang Z. Rong P. Hong Y. Fan Y.. (2016). Transcutaneous vagus nerve stimulation modulates amygdala functional connectivity in patients with depression. J. Affect. Disord. 205, 319–326. 10.1016/j.jad.2016.08.003 27559632

[B39] Lulic D. Ahmadian A. Baaj A. A. Benbadis S. R. Vale F. L. (2009). Vagus nerve stimulation. Neurosurg. Focus 27, E5. 10.3171/2009.6.FOCUS09126 19722820

[B40] Manta S. Dong J. Debonnel G. Blier P. (2009). Enhancement of the function of rat serotonin and norepinephrine neurons by sustained vagus nerve stimulation. J. Psychiatry Neurosci. 34, 272–280. 19568478 PMC2702444

[B41] Masi A. Quintana D. S. Glozier N. Lloyd A. R. Hickie I. B. Guastella A. J. (2015). Cytokine aberrations in autism spectrum disorder: a systematic review and meta-analysis. Mol. Psychiatry 20, 440–446. 10.1038/mp.2014.59 24934179

[B42] McPartland J. C. Reichow B. Volkmar F. R. (2012). Sensitivity and specificity of proposed DSM-5 diagnostic criteria for autism spectrum disorder. J. Am. Acad. Child Adolesc. Psychiatry 51, 368–383. 10.1016/j.jaac.2012.01.007 22449643 PMC3424065

[B43] Ming X. Julu P. O. Brimacombe M. Connor S. Daniels M. L. (2005). Reduced cardiac parasympathetic activity in children with autism. Brain Dev. 27, 509–516. 10.1016/j.braindev.2005.01.003 16198209

[B44] Murphy J. V. Wheless J. W. Schmoll C. M. (2000). Left vagal nerve stimulation in six patients with hypothalamic hamartomas. Pediatr. Neurol. 23, 167–168. 10.1016/S0887-8994(00)00170-3 11020644

[B45] Onore C. Careaga M. Ashwood P. (2012). The role of immune dysfunction in the pathophysiology of autism. Brain Behav. Immun. 26, 383–392. 10.1016/j.bbi.2011.08.007 21906670 PMC3418145

[B46] Park Y. D. (2003). The effects of vagus nerve stimulation therapy on patients with intractable seizures and either Landau-Kleffner syndrome or autism. Epilepsy Behav. 4, 286–290. 10.1016/S1525-5050(03)00080-5 12791330

[B47] Parker A. P. Polkey C. E. Binnie C. D. Madigan C. Ferrie C. D. Robinson R. O. (1999). Vagal nerve stimulation in epileptic encephalopathies. Pediatrics 103(4 Pt 1), 778–782. 10.1542/peds.103.4.778 10103302

[B48] Peuker E. T. Filler T. J. (2002). The nerve supply of the human auricle. Clin. Anat. 15, 35–37. 10.1002/ca.1089 11835542

[B49] Polak T. Markulin F. Ehlis A. C. Langer J. B. Ringel T. M. Fallgatter A. J. (2009). Far field potentials from brain stem after transcutaneous vagus nerve stimulation: optimization of stimulation and recording parameters. J. Neural Transm. 116, 1237–1242. 10.1007/s00702-009-0282-1 19728032

[B50] Porges S. W. (1997). Emotion: an evolutionary by-product of the neural regulation of the autonomic nervous system. Ann. N. Y. Acad. Sci. 807, 62–77. 10.1111/j.1749-6632.1997.tb51913.x 9071344

[B51] Porges S. W. (2001). The polyvagal theory: phylogenetic substrates of a social nervous system. Int. J. Psychophysiol. 42, 123–146. 10.1016/S0167-8760(01)00162-3 11587772

[B52] Porges S. W. (2003). The Polyvagal Theory: phylogenetic contributions to social behavior. Physiol. Behav. 79, 503–513. 10.1016/S0031-9384(03)00156-2 12954445

[B53] Ramsay R. E. Uthman B. M. Augustinsson L. E. Upton A. R. Naritoku D. Willis J.. (1994). Vagus nerve stimulation for treatment of partial seizures: 2. Safety, side effects, and tolerability. First *International Vagus Nerve Stimulation Study Group*. Epilepsia 35, 627–636. 10.1111/j.1528-1157.1994.tb02483.x 8026409

[B54] Rong P. Liu J. Wang L. Liu R. Fang J. Zhao J.. (2016). Effect of transcutaneous auricular vagus nerve stimulation on major depressive disorder: a nonrandomized controlled pilot study. J. Affect. Disord. 195, 172–179. 10.1016/j.jad.2016.02.031 26896810 PMC4828906

[B55] Roosevelt R. W. Smith D. C. Clough R. W. Jensen R. A. Browning R. A. (2006). Increased extracellular concentrations of norepinephrine in cortex and hippocampus following vagus nerve stimulation in the rat. Brain Res. 1119(1): 124–132. 10.1016/j.brainres.2006.08.048 16962076 PMC1751174

[B56] Ruffoli R. Giorgi F. S. Pizzanelli C. Murri L. Paparelli A. Fornai F. (2011). The chemical neuroanatomy of vagus nerve stimulation. J. Chem. Neuroanat. 42, 288–296. 10.1016/j.jchemneu.2010.12.002 21167932

[B57] Sackeim H. A. Keilp J. G. Rush A. J. George M. S. Marangell L. B. Dormer J. S.. (2001). The effects of vagus nerve stimulation on cognitive performance in patients with treatment-resistant depression. Neuropsychiatry Neuropsychol. Behav. Neurol. 14, 53–62. 11234909

[B58] Shiozawa P. Silva M. E. Carvalho T. C. Cordeiro Q. Brunoni A. R. Fregni F. (2014). Transcutaneous vagus and trigeminal nerve stimulation for neuropsychiatric disorders: a systematic review. Arq. Neuropsiquiatr. 72, 542–547. 10.1590/0004-282X20140061 25054988

[B59] Sperling W. Reulbach U. Bleich S. Padberg F. Kornhuber J. Mueck-Weymann M. (2010). Cardiac effects of vagus nerve stimulation in patients with major depression. Pharmacopsychiatry 43, 7–11. 10.1055/s-0029-1237374 20013552

[B60] Steenbergen L. Sellaro R. Stock A. K. Verkuil B. Beste C. Colzato L. S. (2015). Transcutaneous vagus nerve stimulation (tVNS) enhances response selection during action cascading processes. Eur. Neuropsychopharmacol. 25, 773–778. 10.1016/j.euroneuro.2015.03.015 25869158

[B61] Straube A. Ellrich J. Eren O. Blum B. Ruscheweyh R. (2015). Treatment of chronic migraine with transcutaneous stimulation of the auricular branch of the vagal nerve (auricular t-VNS): a randomized, monocentric clinical trial. J. Headache Pain 16:543. 10.1186/s10194-015-0543-3 26156114 PMC4496420

[B62] Tuchman R. Rapin I. (2002). Epilepsy in autism. Lancet Neurol. 1, 352–358. 10.1016/S1474-4422(02)00160-6 12849396

[B63] Warwick T. C. Griffith J. Reyes B. Legesse B. Evans M. (2007). Effects of vagus nerve stimulation in a patient with temporal lobe epilepsy and Asperger syndrome: case report and review of the literature. Epilepsy Behav. 10, 344–347. 10.1016/j.yebeh.2007.01.001 17300990

[B64] Washington S. D. Gordon E. M. Brar J. Warburton S. Sawyer A. T. Wolfe A.. (2014). Dysmaturation of the default mode network in autism. Hum. Brain Mapp. 35, 1284–1296. 10.1002/hbm.22252 23334984 PMC3651798

[B65] Wass S. (2011). Distortions and disconnections: disrupted brain connectivity in autism. Brain Cogn. 75, 18–28. 10.1016/j.bandc.2010.10.005 21055864

[B66] Yirmiya N. Kasari C. Sigman M. Mundy P. (1989). Facial expressions of affect in autistic, mentally retarded and normal children. J. Child Psychol. Psychiatry 30, 725–735. 10.1111/j.1469-7610.1989.tb00785.x 2793960

[B67] Yuan H. Silberstein S. D. (2016). Vagus nerve and vagus nerve stimulation, a comprehensive review: part I. Headache 56, 71–78. 10.1111/head.12647 26364692

[B68] Zablotsky B. Black L. I. Maenner M. J. Schieve L. A. Blumberg S. J. (2014). Estimated Prevalence of Autism and Other Developmental Disabilities Following Questionnaire Changes in the 2014 National Health Interview Survey. U.S. Department of Health and Human Services Centers for Disease Control and Prevention: Natl Health Stat Report (87), 1–20. 26632847

[B69] Zhao Y. X. He W. Gao X. Y. Rong P. J. Zhu B. (2011). Effect of electroacupuncture of auricular concha on inflammatory reaction in endotoxaemia rats. Zhen Ci Yan Jiu 36, 187–192. 21793383

